# Supplementary material for: In vivo single-cell lineage tracing in zebrafish using high-resolution infrared laser-mediated gene induction microscopy
Source: eLife. 2020 Jan 6;9:e52024. doi: 10.7554/eLife.52024 (PMC7018510; doi:10.7554/eLife.52024)
Supplement: Supplementary file 1. — (a) Temperature sensitivity of fluorescent thermometry. (b) Temperature distribution with IR laser heat shock in zebrafish in vivo and efficiency of cell labeling in zebrafish tissues. (c) Numbers of GFP+ T lymphocytes and myeloid cells in each single HE-labeled zebrafish at seven dpf stage. (d) Numbers of GFP+ T lymphocytes and myeloid cells in each non-labeling control zebrafish at seven dpf stage. (e) The estimates and the corresponding 95% asymptotic confidence intervals of the probability for each hemogenic endothelium (HE) lineage in the single-HE labeled group. (f) The estimates and the corresponding 95% asymptotic confidence intervals of the probability for each type of zebrafish in the control group. [file elife-52024-supp1.docx]

**Supplementary File 1a. Temperature sensitivity of fluorescent thermometry**

| Dye | Media/Tissue | TPEF detection ^a^ | Temperature sensitivity (%/$\mathbf{℃}$) | # of measurement |
| --- | --- | --- | --- | --- |
| Individual FITC and TAMRA | FITC solution | fixed point | -0.165±0.0984 | 8 |
|  | TAMRA solution | fixed point | -0.882±0.0997 | 11 |
| Mixed FITC-TAMRA in theoretical model | N/A | N/A | -0.741~-0.794 | N/A |
| Mixed FITC-TAMRA in water and tissue phantom | water | fixed point | -0.843±0.0796 | 7 |
|  | 3% agarose | fixed point | -0.749±0.0424 | 5 |
| Mixed FITC-TAMRA in zebrafish tissues *in vivo* | muscle | scan on 60 µm × 60 µm | -0.753±0.0631 | 9 |
|  | brain | scan on 60 µm × 60 µm | -0.939±0.0687 | 9 |
|  | AGM/PBI ^b^ | scan on 15 µm × 60 µm | -0.762±0.115 | 7 |

^a^ To measure the two-photon excited fluorescence (TPEF) spectra of FITC/TAMRA in water solution and tissue phantom, the excitation laser beam was fixed at a point. To measure the TPEF spectra in zebrafish in vivo, the excitation laser beam was scanned on specific tissues to avoid tissue injury.

^b^ To measure the temperature sensitivity at aorta-gonad-mesonephros (AGM) and posterior blood island (PBI), the excitation laser beam was scanned on a rectangular region (15 µm × 60 µm) to fit the shape of the area.

**Supplementary File 1b. Temperature distribution with IR laser heat shock in zebrafish *in vivo* and efficiency of cell labeling in zebrafish tissues**

| Cell type | Myocyte | | Brain neuron | | Leukocyte ^a^ | |
| --- | --- | --- | --- | --- | --- | --- |
| Heat Shock Condition | High power | Low power | High power | Low power | High  power | Low power |
| IR laser power (mW) | 95 | 80 | 110 | 95 | 100 | 80 |
| Scanning time (s) | 32 | 32 | 32 | 32 | 32 | 32 |
| Scanning area (µm × µm) | 8 × 8 | 8 × 8 | 8 × 8 | 8 × 8 | 8 × 8 | 8 × 8 |
| Temperature at P00 (℃) | 51.3±3.1 | 43.6±3.4 | 48.3±3.5 | 44.6±3.0 | 47.5±4.4 | 40.8±2.2 |
| Temperature at P10 (℃) | 38.5±2.5 | 32.5±1.8 | 42.7±3.0 | 38.0±3.0 | 39.6±3.8 | 32.4±1.1 |
| Temperature at P20 (℃) | 30.5±1.1 | 28.2±1.7 | 36.8±3.6 | 34.7±2.1 | 33.9±2.9 | 29.3±1.7 |
| Total # of zebrafish | 26 | 30 | 21 | 43 | 12 | 18 |
| # of overall-labeled zebrafish | 15 | 11 | 7 | 8 | 9 | 9 |
| # of single-cell labeled zebrafish | 2 | 6 | 4 | 8 | 5 | 7 |
| Success rate of overall cell labeling | 57.7% | 36.7% | 33.3% | 18.6% | 75% | 50% |
| Success rate of single-cell labeling | 13.3% | 54.5% | 57.1% | 100% | 55.6% | 77.8% |

^a^ The *coro1a^+^* leukocytes at aorta-gonad-mesonephros (AGM) and posterior blood island (PBI) were heat shocked.

**Supplementary File 1c. Numbers of GFP^+^ T lymphocytes and myeloid cells in each single HE-labeled zebrafish at 7 dpf stage**

| Fish # | T cell | myeloid cell | Fish # | T cell | myeloid cell | Fish # | T cell | myeloid cell |
| --- | --- | --- | --- | --- | --- | --- | --- | --- |
| fish1 | 8 | 44 | **fish10** | 0 | 8 | **fish19** | 1 | 9 |
| fish2 | 0 | 0 | **fish11** | 16 | 21 | **fish20** | 0 | 3 |
| fish3 | 15 | 8 | **fish12** | 3 | 32 | **fish21** | 0 | 6 |
| fish4 | 0 | 8 | **fish13** | 22 | 45 | **fish22** | 0 | 3 |
| fish5 | 18 | 17 | **fish14** | 0 | 5 | **fish23** | 10 | 14 |
| fish6 | 10 | 40 | **fish15** | 5 | 10 | **fish24** | 0 | 8 |
| fish7 | 0 | 0 | **fish16** | 0 | 7 | **fish25** | 0 | 3 |
| fish8 | 0 | 0 | **fish17** | 2 | 4 | **fish26** | 0 | 4 |
| fish9 | 2 | 25 | **fish18** | 11 | 8 | **fish27** | 0 | 0 |

**Supplementary File 1d. Numbers of GFP^+^ T lymphocytes and myeloid cells in each non-labeling control zebrafish at 7 dpf stage**

| Fish # | T cell | myeloid cell | Fish # | T cell | myeloid cell | Fish # | T cell | myeloid cell |
| --- | --- | --- | --- | --- | --- | --- | --- | --- |
| fish1 | 0 | 0 | **fish38** | 0 | 0 | **fish75** | 0 | 2 |
| fish2 | 0 | 0 | **fish39** | 0 | 0 | **fish76** | 0 | 0 |
| fish3 | 0 | 0 | **fish40** | 0 | 0 | **fish77** | 0 | 6 |
| fish4 | 0 | 0 | **fish41** | 0 | 3 | **fish78** | 0 | 0 |
| fish5 | 0 | 18 | **fish42** | 0 | 0 | **fish79** | 0 | 0 |
| fish6 | 0 | 4 | **fish43** | 0 | 0 | **fish80** | 0 | 2 |
| fish7 | 0 | 0 | **fish44** | 0 | 0 | **fish81** | 0 | 3 |
| fish8 | 0 | 0 | **fish45** | 0 | 1 | **fish82** | 0 | 1 |
| fish9 | 0 | 0 | **fish46** | 0 | 0 | **fish83** | 0 | 0 |
| fish10 | 0 | 1 | **fish47** | 0 | 6 | **fish84** | 0 | 0 |
| fish11 | 0 | 0 | **fish48** | 0 | 0 | **fish85** | 0 | 0 |
| fish12 | 0 | 0 | **fish49** | 0 | 0 | **fish86** | 0 | 2 |
| fish13 | 0 | 0 | **fish50** | 1 | 6 | **fish87** | 17 | 9 |
| fish14 | 0 | 0 | **fish51** | 0 | 3 | **fish88** | 0 | 0 |
| fish15 | 2 | 0 | **fish52** | 0 | 0 | **fish89** | 0 | 3 |
| fish16 | 0 | 5 | **fish53** | 0 | 11 | **fish90** | 0 | 4 |
| fish17 | 6 | 3 | **fish54** | 0 | 5 | **fish91** | 0 | 0 |
| fish18 | 0 | 0 | **fish55** | 14 | 13 | **fish92** | 0 | 0 |
| fish19 | 0 | 2 | **fish56** | 0 | 4 | **fish93** | 0 | 5 |
| fish20 | 0 | 0 | **fish57** | 0 | 0 | **fish94** | 0 | 0 |
| fish21 | 3 | 17 | **fish58** | 0 | 2 | **fish95** | 0 | 1 |
| fish22 | 0 | 0 | **fish59** | 0 | 0 | **fish96** | 0 | 0 |
| fish23 | 0 | 0 | **fish60** | 0 | 6 | **fish97** | 0 | 2 |
| fish24 | 0 | 0 | **fish61** | 0 | 2 | **fish98** | 0 | 0 |
| fish25 | 0 | 0 | **fish62** | 9 | 9 | **fish99** | 0 | 2 |
| fish26 | 0 | 4 | **fish63** | 0 | 0 | **fish100** | 0 | 5 |
| fish27 | 0 | 3 | **fish64** | 0 | 0 | **fish101** | 0 | 4 |
| fish28 | 0 | 3 | **fish65** | 0 | 9 | **fish102** | 0 | 0 |
| fish29 | 0 | 0 | **fish66** | 0 | 2 | **fish103** | 0 | 0 |
| fish30 | 0 | 12 | **fish67** | 0 | 0 | **fish104** | 0 | 0 |
| fish31 | 0 | 0 | **fish68** | 0 | 1 | **fish105** | 0 | 0 |
| fish32 | 16 | 3 | **fish69** | 0 | 0 | **fish106** | 0 | 3 |
| fish33 | 0 | 0 | **fish70** | 0 | 6 | **fish107** | 0 | 2 |
| fish34 | 0 | 26 | **fish71** | 0 | 0 | **fish108** | 13 | 7 |
| fish35 | 0 | 2 | **fish72** | 0 | 2 | **fish109** | 0 | 0 |
| fish36 | 0 | 8 | **fish73** | 0 | 0 |  |  |  |
| fish37 | 0 | 0 | **fish74** | 0 | 0 |  |  |  |

**Supplementary File 1e. The estimates and the corresponding 95% asymptotic confidence intervals of the probability for each hemogenic endothelium (HE) lineage in the single-HE labeled group**

| Lineage | Probability | Lower limit | Upper limit |
| --- | --- | --- | --- |
| Lymphoid & Myeloid | **0.4379** | 0.3863 | 0.4896 |
| Lymphoid | **0.0000** | 0.0000 | 0.0687 |
| Myeloid | **0.2841** | 0.2587 | 0.3095 |
| Others | **0.2780** | 0.2546 | 0.3014 |

**Supplementary File 1f. The estimates and the corresponding 95% asymptotic confidence intervals of the probability for each type of zebrafish in the control group**

| ɛ ^a^ | Probability | Estimate | Lower limit | Upper limit |
| --- | --- | --- | --- | --- |
| (+,+) | ***q*_11_** | **0.0734** | 0.0692 | 0.0776 |
| (+,0) | ***q*_10_** | **0.0086** | 0.0072 | 0.0100 |
| (0,+) | ***q*_01_** | **0.3944** | 0.3858 | 0.4031 |
| (0,0) | ***q*_00_** | **0.5236** | 0.5145 | 0.5326 |

^a^ $\varepsilon=(\varepsilon_{T},\varepsilon_{M})$ is the sign of the number of T or M cells in a non-labeling zebrafish in the control group.
